# When Forks Came in

**Published:** 1892-11

**Authors:** 


### WHEN FORKS CAME IN.

It was about the year 1600, and in the reign of James I., when forks where first introduced into England. This "piece of refinement," we are told, was derived from the Italians. In a curious book of travels, published in the year 1611, the writer says :

I observed a custom in all those Italian cities and towns through the which I passed that is not used in any other country that I saw in my travels. Neither do I think that any other nation in Christendome doth use it, but only Italy. The Italians, and also most strangers that are commorant in Italy, do alwaies at their meales use a little forke, when they cut their meate. For while with their knife, which they hold in one hand, they cut the meate out of the dish, they fasten their forke, which they hold in the other hand, upon the same dish. This form of feeding is generally in use in all Italy, their forkes being for the most part made of yron, or steel, and some of silver, but those are used only by gentlemen." Before the revolution in France it was customary when a gentleman had been invited out to dinner, to send his servant in advance with his knife, fork, and spoon. If he had no servant he carried them with him in his pocket. Some of the peasantry in certain parts of Germany and Switzerland to-day carry a case in their pockets, containing a knife, fork, and spoon.

